# Probiotic Supplementation With Saccharomyces boulardii and Enterococcus faecium Improves Gastric Pain and Bloating in Airline Pilots With Chronic Non-atrophic Gastritis: An Open-Label Study

**DOI:** 10.7759/cureus.52502

**Published:** 2024-01-18

**Authors:** Piercarlo Minoretti, Miryam Liaño Riera, Andrés Santiago Sáez, Manuel Gómez Serrano, Ángel García Martín

**Affiliations:** 1 General Direction, Studio Minoretti, Oggiono, ITA; 2 Legal Medicine, Psychiatry and Pathology, Complutense University of Madrid, Madrid, ESP; 3 Legal Medicine, Hospital Clinico San Carlos, Madrid, ESP

**Keywords:** enterococcus faecium, saccharomyces boulardi, probiotics, airline pilots, chronic non-atrophic gastritis

## Abstract

Background

Commercial airline pilots (APs) are prone to upper gastrointestinal symptoms, such as epigastric pain and bloating. These issues are often linked to occupational risk factors like irregular diet, sleep disruption, and circadian rhythm disturbance. The use of probiotics to enhance intestinal health is well established, but their efficacy in treating upper gastrointestinal diseases is still debated. This is primarily due to the stomach’s small resident microbiota and its low pH, which is inhospitable to most microbes. However, emerging research suggests that specific probiotic strains, such as *Enterococcus faecium*, can withstand acidic environments. Moreover, certain yeast species, including *Saccharomyces boulardii*, can survive at a low pH. Consequently, we conducted a preliminary, three-arm, randomized, open-label, dose-finding, four-week study to compare the effects of watchful waiting (WW) with the administration of an oral probiotic supplement containing *S. boulardii *and *E. faecium* in APs diagnosed with *Helicobacter pylori*-negative chronic non-atrophic gastritis (CNG).

Methods

The study included 39 APs with CNG who were randomized into three groups with a 1:1:1 ratio. The low-dose group (n = 13) received one capsule of the probiotic supplement twice daily, before meals, for four weeks. The high-dose group (n = 13) was administered two capsules of the supplement on the same schedule. The third group (n = 13) underwent WW and served as the control arm. Blinding was maintained for the examining physicians and laboratory staff, but not for the patients. All participants self-rated their experiences of gastric pain and bloating at the beginning and conclusion of the four-week treatment period. Additionally, serum levels of pepsinogen I (PGI) and pepsinogen II (PGII) were measured at these time points.

Results

Supplementation with probiotics significantly outperformed WW in reducing subjective gastric pain and bloating. This effect was consistent across both tested dosages, with no significant differences observed. However, only high-dose probiotics led to a statistically significant decrease in PGII levels and an increase in the PGI/PGII ratio after the four-week study period, a result not observed with low-dose probiotics.

Conclusions

Oral administration of *S. boulardii* and *E. faecium *demonstrated potential efficacy in reducing gastric pain and bloating symptoms in APs with CNG, as evidenced by statistically significant symptom improvement compared to the control group that did not receive the probiotic supplementation. Notably, high-dose probiotics resulted in a significant increase in the PGI/PGII ratio, indicating potential long-term cytoprotective effects on the gastric mucosa.

## Introduction

Commercial airline pilots (APs) are prone to upper gastrointestinal symptoms, such as epigastric pain and bloating. These issues are often linked to occupational risk factors like irregular diet, sleep disruption, and circadian rhythm disturbance [1]. A study conducted on 354 APs working for a Swedish airline company revealed that 15.2% suffered from heartburn, 62.1% experienced bloating, and 14.4% reported epigastralgia [2]. Interestingly, all three symptoms were found to be associated with insomnia [2]. Another study involving 212 male APs in a Chinese large civil airline company reported a prevalence of 39.22% for functional gastrointestinal disorders [3]. The main independent risk factors included flight level, high-salt food patterns, and sleep performance [3]. In a recent seroprevalence study conducted on 100 male APs, we found that previous infections with *Helicobacter pylori* were significantly more common among APs compared to a control group consisting of office workers [4].

Chronic gastritis, a common condition characterized by long-term inflammation of the stomach mucosa, is influenced by a multitude of factors, both occupational and non-occupational [5,6]. This condition can be categorized into chronic non-atrophic gastritis (CNG), chronic atrophic gastritis (CAG), and other specific types of gastritis [7]. CNG, marked by chronic inflammatory cell infiltration and no atrophy in the mucosal layer, constitutes about half of all chronic gastritis cases [8,9]. Treatment for CNG typically involves medication, dietary changes, and the removal of risk factors such as smoking, alcohol consumption, and chronic use of non-steroidal anti-inflammatory drugs [5]. Proton pump inhibitors, which inhibit gastric acid secretion, are a primary treatment. However, long-term acid suppression can hasten the loss of gastric glandular structures, potentially leading to a transition from CNG to CAG [8]. In cases where patients with CNG do not see improvements with standard long-term management, alternative approaches to conventional therapies may be considered [9-11].

The use of probiotics to enhance intestinal health is well established, but their efficacy in treating upper gastrointestinal diseases is still debated [12]. This is primarily due to the stomach’s small resident microbiota and its low pH (between 2.5 and 3.5), which is inhospitable to most microbes [12]. However, emerging research suggests that specific probiotic strains, such as *Enterococcus faecium*, can withstand acidic environments [13]. Moreover, certain probiotic yeast species, including *Saccharomyces boulardii*, can survive at a pH of 3.0 [14]. Consequently, we conducted a preliminary, three-arm, randomized, open-label, dose-finding, four-week study to compare the effects of watchful waiting (WW) with the administration of an oral probiotic supplement containing *S. boulardii* and *E. faecium* in APs diagnosed with* H. pylori*-negative CNG. We also measured serum levels of pepsinogen I (PGI) and pepsinogen II (PGII), two peptides originating from the gastric mucosa [15], as biochemical markers of response to probiotic supplementation.

## Materials and methods

Participants

This study represents an exploratory, three-arm, randomized, open-label, dose-finding investigation conducted over a four-week period, involving 39 male APs (mean age: 43.4 ± 2.5 years) diagnosed with CNG. The research was carried out in an occupational medicine setting at outpatient facilities located at Studio Minoretti SRL (Oggiono, Italy) [4,16,17]. The inclusion criteria were as follows: (1) a confirmed diagnosis of CNG based on endoscopic and histopathological evidence [7]; (2) an age range between 18 and 65 years; and (3) willingness to participate in the study. All APs tested negative for *H. pylori* via stool antigen tests. The following exclusion criteria were applied: (1) history of gastric surgery; (2) gastritis differing from CNG, including CAG and other specific types of gastritis; (3) peptic ulcer and gastric hemorrhage; (4) dysplasia of gastric mucosa; (5) cholecystitis and cholelithiasis; (6) severe primary diseases of the cardiovascular system, cerebrovascular system, lung, liver, kidney, and hematopoietic system; (7) aspartate aminotransferase (AST) and/or alanine transaminase (ALT) ≥ 1.5 × upper limit of normal (ULN) and/or creatinine ≥ 1.3 × ULN; (8) use of drugs to treat CNG in the two weeks preceding the study; and (9) atopy or allergic disorders. Moreover, women were not considered eligible because the sample was too small. The study was approved by Studio Minoretti SRL (approval number: 2022/PRO/AP). It was conducted in accordance with the principles of the Declaration of Helsinki. Each participant was thoroughly informed about the study objectives, and written consent was obtained.

Probiotic supplement

The probiotic supplement used in this study (Enteroboulardi; Laboratori Legren, Imperia, Italy) was procured from local pharmacies. The supplement was in capsule form, with one capsule containing *S. boulardii* (3 billion) and *E. faecium* (1 billion). The supplement was chosen from a range of commercially available probiotics due to the anticipated resistance of both *S. boulardii* [14] and *E. faecium* [13] to low pH environments.

Procedures

APs with CNG (n = 39) were randomly divided into three groups (1:1:1 ratio). The random allocation sequence was generated by a computer program. The low-dose group (n = 13) received one capsule of the probiotic supplement twice daily, before meals, for four weeks. The high-dose group (n = 13) was administered two capsules of the supplement on the same schedule. The third group (n = 13) underwent WW and served as the control arm. Examining physicians and laboratory personnel were blinded to group assignment, whereas APs were unblinded. All participants were asked to suspend other treatments during the study course. Two assessments were conducted: one at baseline and one at the end of the four-week supplementation period.

Clinical endpoints

The clinical endpoints evaluated at the study initiation and after the four-week supplementation period included (1) the patient’s self-assessment of gastric pain using a visual analogue scale (VAS), with a range from 0 (no pain) to 10 (worst possible pain) [18] and (2) the patient’s self-assessment of bloating based on item 3 of the Patient Assessment of Constipation-Symptoms (PAC-SYM) questionnaire, with a range from 0 (no bloating) to 4 (very severe bloating) [19].

Measurements of serum levels of PGI and PGII

Venous blood samples were collected using serum separator tubes during each assessment. The collected blood was allowed to clot at room temperature for 30 minutes, followed by centrifugation at 1,000 × g for 15 minutes. After centrifugation, the serum was carefully separated, and aliquots were immediately frozen at −80°C until further analysis. Serum PGI and PGII levels were quantified using chemiluminescent enzyme immunoassays (ARCHITECT Pepsinogen I and II Reagent Kits; Abbott Laboratories Inc., Chicago, IL, USA). The assays were performed in accordance with the manufacturer’s protocol. All samples were processed simultaneously at the conclusion of the study by laboratory personnel who were blinded to the clinical data. Each subject’s specimens were analyzed in duplicate within the same assay.

Safety measures

Safety was evaluated by documenting any adverse events that emerged during treatment and noting any changes from the initial baseline in clinical laboratory tests, vital signs, and physical examinations. All assessments were conducted at the start of the study and upon its completion. For clinical laboratory tests, we considered changes from baseline to be clinically relevant if they met the following criteria [20]: an increase in AST and/or ALT to three times or more of the ULN; an increase in creatinine to 1.3 times or more of the ULN; an increase in blood urea nitrogen to twice or more of the ULN; a decrease in hematocrit by 5 percentage points or more from the baseline; and a decrease in hemoglobin by 2 g/dL or more from the baseline.

Data analysis

Categorical variables, presented as counts and percentage frequencies, were analyzed using the chi-square test. Continuous data were expressed as means ± standard deviations and compared using a one-way analysis of variance, followed by *post hoc* Newman-Keuls tests. All calculations were performed using IBM SPSS Statistics for Windows, Version 20.0 (Released 2011; IBM Corp., Armonk, NY, USA). Two-tailed P-values < 0.05 were considered statistically significant.

## Results

Baseline characteristics

The baseline characteristics of APs in the three study groups are summarized in Table 1. There were no significant intergroup differences in terms of age, sex, body mass index, VAS for gastric pain, or bloating scores, suggesting that randomization was performed properly. Laboratory safety parameters (AST, ALT, creatinine, BUN, hematocrit, and hemoglobin) at baseline were all within the normal range (data not shown). The study sample may therefore be considered representative of APs with CNG in need of clinical management.

**Table 1:** Baseline characteristics of the three study groups ns, not significant; VAS, visual analogue scale

Variable	Watchful waiting (n = 13)	Supplementation with low-dose probiotics (n = 13)	Supplementation with high-dose probiotics (n = 13)	P
Age, years	43.2 ± 2.4	43.5 ± 2.6	43.3 ± 2.5	ns
Men, n (%)	13 (100%)	13 (100%)	13 (100%)	ns
Body mass index, kg/m^2^	25.2 ± 2.4	24.8 ± 2.6	24.9 ± 2.9	ns
Gastric pain, VAS (0-10)	5.7 ± 0.9	5.9 ± 0.9	5.8 ± 1.0	ns
Bloating (0-4)	1.9 ± 0.6	1.8 ± 0.7	1.9 ± 0.7	ns

Clinical endpoints

No AP withdrew from the study. The clinical endpoints in the low- and high-dose probiotic groups are shown in Table 2. As expected, APs in the WW group did not exhibit significant changes over the course of the study. Compared to baseline values, the probiotic supplement significantly reduced both the VAS for gastric pain (P < 0.01) and bloating scores (P < 0.05) at four weeks in both the low-dose and high-dose groups, with both dosages showing similar reductions compared to baseline values.

**Table 2:** Temporal course of clinical endpoints in the three study groups The data are expressed as means and standard deviations. *P < 0.05 versus baseline; **P < 0.01 versus baseline VAS, visual analogue scale

Clinical endpoint	Watchful waiting (n = 13)		Low-dose probiotics (n = 13)		High-dose probiotics (n = 13)	
	Baseline	End of the study	Baseline	End of the study	Baseline	End of the study
Gastric pain, VAS (0-10)	5.7 ± 0.9	5.8 ± 1.1	5.9 ± 0.9	4.9 ± 1.1**	5.8 ± 1.0	4.7 ± 1.2**
Bloating (0-4)	1.9 ± 0.6	1.8 ± 0.7	1.8 ± 0.7	1.4 ± 0.4*	1.9 ± 0.7	1.5 ± 0.6*

Serum levels of PGI and PGII

Table 3 depicts the temporal variations in serum PGI and PGII levels in the three study groups. At baseline, there were no significant intergroup differences in terms of PGI and PGII levels, as well as the PGI/PGII ratio. At four weeks, high-dose probiotics, but not low-dose probiotics or WW, produced statistically a significant reduction in PGII levels (P < 0.01), which was paralleled by an increase in the PGI/PGII ratio (P < 0.01) compared with baseline values. These differences between the low- and high-dose groups at the end of the four-week study period were statistically significant (both P < 0.01).

**Table 3:** Temporal course of serum PGI and PGII levels in the three study groups **P < 0.01 versus baseline; †P < 0.01 versus supplementation with low-dose probiotics PGI, pepsinogen I; PGII, pepsinogen II

Serum biomarker	Watchful waiting (n = 13)		Supplementation with low-dose probiotics (n = 13)		Supplementation with high-dose probiotics (n = 13)	
	Baseline	End of the study	Baseline	End of the study	Baseline	End of the study
Serum PGI levels, ng/mL	94.8 ± 12.5	95.1 ± 10.4	96.6 ± 10.7	91.7 ± 8.1	92.8 ± 15.9	87.2 ± 16.2
Serum PGII levels, ng/mL	10.8 ± 1.3	10.5 ± 1.1	10.5 ± 1.2	10.3 ± 1.0	10.8 ± 1.5	8.8 ± 1.3**,†
PGI/PGII ratio	8.7 ± 1.2	9.0 ± 1.0	9.2 ± 1.3	8.9 ± 1.2	8.5 ± 1.5	9.9 ± 1.2**,†

Safety

Treatment-emergent adverse events were sporadic and did not differ significantly in the three study groups (Table 4). Clinically relevant changes in laboratory values did not occur in any of the study participants, regardless of the treatment arm (data not shown).

**Table 4:** Treatment-emergent adverse events observed in the three study groups

Adverse event	Watchful waiting (n = 13)	Supplementation with low-dose probiotics (n = 13)	Supplementation with high-dose probiotics (n = 13)
Headache	0 (0%)	1 (7.7%)	1 (7.7%)
Dizziness	0 (0%)	1 (7.7%)	0 (0%)
Pruritus	0 (0%)	0 (0%)	1 (7.7%)

## Discussion

In this exploratory study, the administration of *S. boulardii* and *E. faecium* demonstrated potential efficacy in reducing gastric pain and bloating symptoms in APs with CNG, as evidenced by statistically significant symptom improvement compared to the control group that did not receive the probiotic supplementation. The decrease in self-rated experiences of gastric pain and bloating was consistent across different dosages. However, only the high-dose probiotics led to a statistically significant reduction in PGII levels and an increase in the PGI/PGII ratio after the four-week study period. Treatment-emergent adverse events were rare throughout the supplementation period, and none resulted in participant withdrawal. Furthermore, no significant changes were detected in clinical laboratory safety tests for either dosage group.

Non-pharmacological strategies for managing CNG are gaining increased attention [9,10]. The probiotic yeast *S. boulardii* is recognized for its resilience to low pH environments [14]. Notably, it has demonstrated the ability to remain viable even after exposure to simulated gastric juice, which contains pepsin and hydrochloric acid, thus enabling it to survive in stomach-like conditions [21]. Similarly, *E. faecium* exhibits resistance to low pH and is unaffected by bile salts [13,22], unlike most bacteria, which are unable to survive under such conditions. The unique ability of these two probiotics to withstand the harsh gastric environment has led us to investigate their combined use for CNG. However, the exact mechanisms by which *S. boulardii* and *E. faecium* may alleviate symptoms such as gastric pain and bloating, as observed in our study, remain speculative. One hypothesis is that *S. boulardii* can influence immunological function by enhancing the synthesis of short-chain fatty acids, including butyrate [23]. This is of interest as butyrate may aid in the repair of the gastric mucosa due to its anti-oxidative and anti-inflammatory properties [24]. In addition, animal studies have demonstrated that *E. faecium* may exert an immunomodulatory function [25]. This includes the suppression of pro-inflammatory cytokines, stimulation of the expression of anti-inflammatory cytokines, and stabilization of the expression of toll-like receptor genes [26].

Persistent bloating, often described as a feeling of trapped gas and fullness, is a prevalent symptom among patients with CNG [5]. This discomfort can be attributed to disordered gastric motility and visceral hypersensitivity [19]. Interestingly, our study observed a significant reduction in bloating after probiotic supplementation, which could potentially be linked to the presence of *E. faecium*. This bacterium is capable of producing dopamine in environments similar to the gastrointestinal tract, thereby influencing the gastrointestinal dopaminergic pathways [27]. Notably, gastric epithelial cells possess dopaminergic receptors [28], and dopamine agonists can enhance the stomach secretion of protective mucus and bicarbonate [29]. Furthermore, dopamine can modulate gastric motility [28], providing a plausible explanation for the observed decrease in bloating complaints among APs with CNG who were administered a probiotic containing *E. faecium*.

While the two tested dosages were equally effective in relieving gastric pain and bloating, only high-dose probiotics resulted in a significant decrease in serum levels of PGII and an increase in the PGI/PGII ratio. PGI is primarily secreted by mucosal cells in the fundus, whereas PGII is predominantly secreted by chief cells, the pyloric glands, and the proximal duodenal mucosa [30]. An increase in PGII concentration, accompanied by a decrease in the PGI/PGII ratio, is indicative of the severity of gastric inflammation [30]. Interestingly, a consistent decrease in the PGI/PGII ratio has been linked to the progression from normal gastric mucosa to precancerous gastric lesions [31]. Taken together, these findings suggest that high-dose probiotics, despite having similar short-term clinical effectiveness as low-dose probiotics, may potentially offer long-term cytoprotective effects on the gastric mucosa that are not provided by the low-dose supplementation scheme.

This study has several limitations. The small sample size may lead to an overestimation of treatment effects. In addition, the use of continuous endpoints and self-reported outcomes could introduce potential bias [20]. To mitigate these issues, we incorporated objectively measured biochemical endpoints, specifically serum PGI and PGII levels. This study should be viewed as an exploratory analysis. No endoscopic examinations were conducted, the follow-up period was brief, and independent replication is necessary to validate and expand our findings. Importantly, our study was conducted within a highly specialized context, specifically occupational medicine, for a unique professional group such as APs, who are exposed to highly specific gastrointestinal risk factors. Finally, our decision to employ WW as a control arm rather than a placebo was influenced by two primary factors. Firstly, the probiotic combination utilized in our research was commercially available, making it unfeasible for our team to fabricate a placebo that would be physically indistinguishable from the study product. Secondly, we were operating under financial limitations, and the execution of a placebo-controlled study would have incurred significantly higher expenses.

## Conclusions

Despite the limitations, our preliminary findings suggest that oral administration of *S. boulardii* and *E. faecium* demonstrated potential efficacy in reducing gastric pain and bloating symptoms in APs with CNG, as evidenced by statistically significant symptom improvement compared to the control group that did not receive the probiotic supplementation. While the decrease in self-rated experiences of gastric pain and bloating was consistent across different dosages, high-dose probiotics resulted in a significant increase in the PGI/PGII ratio, indicating potential long-term cytoprotective effects on the gastric mucosa. However, these results should be interpreted as hypothesis generating. In general, treatment for patients with CNG should be individualized, taking into account the unique characteristics of their disease and the most recent clinical trial data, irrespective of their profession.
